# How Patients Contribute to an Online Psychoeducation Forum for Bipolar Disorder: A Virtual Participant Observation Study

**DOI:** 10.2196/mental.4123

**Published:** 2015-07-08

**Authors:** Ria Poole, Daniel Smith, Sharon Simpson

**Affiliations:** ^1^ Centre for the Development and Evaluation of Complex Interventions for Public Health Improvement (DECIPHer) School of Social Sciences Cardiff University Cardiff United Kingdom; ^2^ Mental Health and Wellbeing University of Glasgow Glasgow United Kingdom; ^3^ MRC/CSO Social and Public Health Sciences Unit University of Glasgow Glasgow United Kingdom

**Keywords:** bipolar disorder, psychoeducation, Internet, forum, qualitative

## Abstract

**Background:**

In a recent exploratory randomized controlled trial, an online psychoeducation intervention for bipolar disorder has been found to be feasible and acceptable to patients and may positively impact on their self-management behaviors and quality of life.

**Objective:**

The objective of the study was to investigate how these patients contribute to an online forum for bipolar disorder and the issues relevant for them.

**Methods:**

Participants in the intervention arm of the Bipolar Interactive PsychoEDucation (“BIPED”) trial were invited to contribute to the Beating Bipolar forum alongside receiving interactive online psychoeducation modules. Within this virtual participant observation study, forum posts were analyzed using thematic analysis, incorporating aspects of discourse analysis.

**Results:**

The key themes which arose from the forum posts included: medication, employment, stigma, social support, coping strategies, insight and acceptance, the life chart, and negative experiences of health care. Participants frequently provided personal narratives relating to their history of bipolar disorder, life experiences, and backgrounds, which often contained emotive language and humor. They regularly sought and offered advice, and expressed encouragement and empathy. The forum would have benefitted from more users to offer a greater support network with more diverse views and experiences.

**Conclusions:**

Online forums are inexpensive to provide and may offer peer support and the opportunity for patients to share their experiences and explore issues related to their illness anonymously. Future research should focus on how to enhance patient engagement with online health care forums.

**Trial Registration:**

ISRCTN81375447; http://www.isrctn.com/ISRCTN81375447 (Archived by WebCite at http://www.webcitation.org/6YzWtHUqu).

## Introduction

### Bipolar Disorder: Isolation, Social Conflict, and Self-Doubt

Many people with long-term conditions may feel isolated if they do not know others who have the same condition or if their condition has impacted adversely on their work and social life [1-3]. The latter is commonly the case for people with bipolar disorder, as their families, friends, and colleagues may not be able to cope with their mood swings or the impact of them [2]. A qualitative study of people with bipolar disorder by Michalak et al found that many interviewees reported that they had lost relationships with partners, friends, and family members as a direct result of their bipolar disorder, particularly during hypomanic and manic episodes [4]. Another study found that the lives of many people with bipolar disorder were characterized by disruption, confusion, contradiction, and self-doubt, and consequently stressed the importance of interventions, which facilitate acceptance [5].

### Internet-Based Psychoeducation

Group psychoeducation enables people to meet with others who have the same health condition, whereas Internet-based psychoeducation may deliver social support through online forums or email [6,7]. Such peer support may offer empathy and advice through shared experiences, help others to understand and come to terms with stressful life events, and provide effective coping strategies and signposting to helpful resources [3,8,9].

Internet resources, which provide health information, are increasing in number and popularity [10,11]. Accessing Internet health information has an empowering effect, as patients and caregivers take an active role in managing their health and receiving peer support [10]. “Expert patients” manage their condition by developing knowledge relevant to managing their health [12] and making informed decisions regarding their treatment [13]. A survey of 3001 adults in the United States revealed the following statistics for the 74% of adults surveyed who used the Internet [11],

- 34% had read someone else’s commentary or experience about health or medical issues on an Internet news group, website, or blog [11];
- 18% had gone on to the Internet to find others who might have health concerns similar to theirs [11];
- 6% had posted comments, questions, or information about health or medical issues on a website [11]; and
- 4% had posted their experiences with a particular drug or medical treatment [11].

Although there is an understanding of trends in seeking health information on the Internet in broad terms, research upon the use of online discussion forums for people with bipolar disorder is minimal [1,14,15]. A German study analyzed two forums for patients with bipolar disorder, examining 1200 contributions of 135 users, according to “fields of interest” and “self-help mechanisms” [14]. The authors found that patients mostly discussed their social networks, symptoms of the illness, and medication, primarily in order to share their emotions [14]. They also identified disclosure, group cohesion, empathy, and support to be the main self-help mechanisms [14].

A Spanish study of an online forum for bipolar disorder focused solely on exploring contradictions between the first posts of a new user and other member’s replies giving unsolicited advice [15]. The authors used conversation analysis to examine the sequential features of communication [15]. The main finding from this study was that there was commonly an apparent mismatch between what the new user appealed for and the responses given by other users [15]. New users who sought accounts of others’ experiences, reassurance, or basic information were given unsolicited advice by existing members [15], which the authors interpret as being instructive and a way of asserting the culture of the group.

Cultural differences may account for some differences between the ways users of both studies typically communicate within the forums. To our knowledge, no research has been conducted into how British patients use a forum for bipolar disorder. We sought to explore participants’ contributions to an online psychoeducation forum, which was part of the Bipolar Interactive PsychoEDucation (“BIPED”) trial [16]. There were twenty-four participants who were allocated to the intervention arm of the trial that were provided with user accounts to access the forum. Dr Smith moderated the forum and all users could initiate forum thread topics. This qualitative study aimed to explore contributions to this forum during the 14 weeks within which participants accessed the Beating Bipolar psychoeducation modules. We identify topics that individuals with bipolar disorder raised or discussed in the forum, which seemed to be important or relevant to them, and report how participants engaged with the forum and with other users.

## Methods

### Design and Recruitment

This virtual observation study was carried out for the evaluation of the phase II randomized controlled BIPED trial, trial registration number ISRCTN81375447, approved by the South East Wales National Health Service Research Ethics Committee [9].

Research into computer-mediated communication (CMC) has shifted in its epistemological focus from viewing CMC as a research “tool” [17] to recognizing CMC as a site of investigation and a culture to be explored [18,19]. As an adaptable methodology appropriate for the study of online communities and cultures, “virtual participant observation” [20] (also referred to as “online ethnography”, “netnography”, and “virtual ethnography”) is increasingly used within many disciplines; including sociology, philosophy, psychology, and economics [17,19,21,22]. To reflect the values of ethnography, proponents of virtual participant observation state the importance of providing a Geertzian “thick description” [14] through immersing the researcher in the life of the online community or culture [8,9]. This immersion in the life of the community may be achieved through directly participating in an online forum or through combining different research methods [15], to include interviews or focus groups, for example.

In this study, DS “announced” his online presence within the online forum as “Dr Smith”, who was known to participants of the BIPED trial as a psychiatrist and a researcher of the Beating Bipolar psychoeducation program. DS contributed posts within the forum to initiate topics for discussion, and in this sense became immersed within the online community as a participatory member, in the sense that he took part in the forum on a two week basis. Participants were invited to contribute to the online forum alongside receiving the eight Beating Bipolar psychoeducation online modules on a two week basis.

Findings from this virtual observation study are also considered in relation to findings from qualitative interviews with the same Beating Bipolar participants [2] to more fully understand how patients contribute to the forum and the barriers and facilitators to them doing so.

### Data Collection and Analysis

Forum usage data were obtained from the software company who created a database to record this information to explore how many users posted contributions or created new topic threads and how often. Written data were extracted from the forum into a text document, which was consequently uploaded to the qualitative analysis software program NVivo 8.

To study the way participants used language to convey meaning and construct their identities, discourse analysis [23] was used in conjunction with thematic analysis [24], the latter chosen because of its flexibility and applicability to various types of data and theoretical frameworks.

Discourse analysis examines text or spoken language to identify underlying social structures, which may be implied through metaphors, word choice, or speech patterns for emphasis, for example [25]. Discourse analysis is intrinsically linked to thematic analysis; linguistic considerations are taken into account as the researcher analyzes the data for recurring themes and categories. According to discourse analysts, discourse pertains to themes that relate to identity in particular [25]. In the present study, we wished to explore how identity is constructed within the group of Beating Bipolar forum users in terms of how they interact with each other and what they discuss as being important to them. We conducted thematic analysis of forum posts, which also considered patients’ discourse in terms of the language they used to convey meaning in the experiences they described.

A mostly inductive approach to analysis was chosen, whereby themes were identified as they emerged from the data, rather than being driven by the headings of the topic threads. We conducted thematic analysis as described by Braun and Clarke [24]. Themes occurred as patterned responses within the data. The coding framework developed in a responsive manner to the themes elicited within the forum posts, and patterns within and across themes were explored throughout the analytic process. There were three members of the research team that read the forum data (DS, RP, and KW) for initial impressions. KW assisted with data analysis for his project as an undergraduate medical student. DS and RP made notes of our impressions of the forum, which facilitated reflexivity, orientation to, and immersion in the data. RP developed an initial coding framework for data analysis when reading through the forum posts prior to coding using NVivo. This framework was discussed with the team prior to conducting in-depth analysis, for which it provided the structural ground for coding; the framework was inputted into NVivo as parent nodes (or top-level headings) with child nodes (potential subcategories, which were subject to alterations as coding proceeded) beneath. Top level headings for emerging themes within the coding framework were: “What do people say?”, “How do people say it?”, and “How do people engage with others?”. RP and KW independently coded the data according to the coding framework, which was developed and refined through discussion during the analytic process. Hence, the whole dataset was double coded for consistency and agreement of interpretation for emerging themes. Where there were any uncertainties, consensus was achieved through discussion. We identified the main themes and subthemes, and interpreted users’ interactions with each other.

Participants of the trial consented for us to use results from the forum data within our research; however, at the point of obtaining informed consent, we did not specifically state that we would use quotes from the forum to illustrate our research findings, and therefore we have chosen not to include participants' direct quotes when reporting results.

## Results

### Participation Within the Forum

Of the 24 participants who were provided with exclusive access to the forum 13 (54%) contributed at least once to the forum and 10 (42%) created a new topic for discussion. There were one hundred and twenty-seven posts that were generated in total, 92 (72.4%) of which were contributed by four participants (4/24) (16%) who dominated the forum. Table 1 presents the number of posts per participant who contributed to the forum.

**Table 1:** Number of posts per participant who contributed to the forum.

Participant identification number	Gender	Number of posts
1	Female	16
2	Male	46
3	Female	15
4	Female	4
5	Female	11
6	Male	7
7	Male	8
8	Male	3
9	Female	1
10	Female	5
11	Male	6
12	Male	4
13	Female	1

### Key Themes

The key themes identified within the analysis were: medication, employment, social stigma, social support, coping strategies, insight and acceptance, the life chart, and negative experiences of health care. Please note that PID refers to the participant identification number; for example, PID1 is Participant 1.

### Medication

Medication was the predominant topic for discussion. There were 44/127 posts (34.6%) that related to medication. Participants mostly discussed the side effects of medication from their personal experiences and the trial and error process of finding the right combination of medication. Many participants described their experiences with lithium, and weight gain was a particular concern.

PID1 said that after 15 years she has now come to terms with the illness and takes lithium “religiously”. She tries to ignore the side effects because without the medication she feels she would be ill again. PID2 responded to this post to say that he felt encouraged by this person’s experience of lithium and would start a new topic thread for people to share their experiences of different combinations of medication.

PID5 participant said that she put on a lot of weight and became really lethargic when taking lithium for six years and felt very unhappy. A couple of participants commented that despite the side effects, being on lithium enabled them to lead a balanced life.

As a result of viewing the medication module, PID12 reported feeling frustrated that his doctor would only prescribe him antidepressants in spite of the fact that he doesn’t respond well to them.

### Employment

Employment was the next most popular topic for discussion, with 30/127 posts (23.6%). Participants mostly expressed difficulty in securing or holding down a job. Stigma regarding mental health issues was noted by a number of participants, and some participants gave personal accounts of prejudice or discrimination in the workplace. Advice was sought regarding how to get a job, and many expressed their frustrations and dissatisfaction with being unemployed or with their current job. Boredom, self-esteem, and financial issues were key subthemes.

PID11 said that he lost three jobs as a result of his behavior during manic episodes. PID3 remarked that she had to give up a very well paying job because of the illness. Some participants commented that their careers have ended due to their bipolar disorder, and sought advice from other forum users regarding potential work opportunities.

Some participants remarked upon the issue of explaining gaps in their employment histories. PID10 tried to hide her bipolar disorder from her employer for 15 years. PID2 complained that in his experience, employers do not risk employing a person with bipolar disorder because they cannot afford to cover months of sick leave.

PID9 commented that she felt that her only way back into work would be via the voluntary sector. She expressed a desire to do something to stimulate her brain again and give her life purpose. Another participant recommended doing administrative work, because it had improved her self-esteem, confidence, and Curriculum Vitae.

The Disability Discrimination Act was cited by a couple of participants. PID7 remarked that although legally employers need to make adjustments for the condition, what happens in practice might vary.

### Social Stigma

Stigma was a key theme that pervaded many topic threads. Participants discussed how others perceive bipolar disorder. The portrayal of bipolar disorder in the media was discussed, and participants felt that more accurate examples in the media may improve public awareness of bipolar disorder and reduce social stigma. Participants expressed their fear of disclosing their bipolar disorder, and some reported concealing their illness from others because of stigma. Some felt stigmatized by friends and family, insecure and ashamed of themselves.

PID5 revealed that she wouldn’t disclose her bipolar disorder to anyone other than close friends and family because of her feeling that others have preconceptions, misunderstandings, or stereotypes of the condition. PID7 reported feeling stigmatized and misunderstood by his family and friends.

PID3 said that she was told not to tell anyone about her bipolar disorder because of the stigma, the possibility of losing her job, and having her children taken into care. She reported feeling dazed, frightened, insecure, and ashamed.

Some participants recommended television screenings that address the issue of stigma surrounding mental health. Some participants mentioned the celebrity Stephen Fry, regarding bringing the issue of bipolar disorder into public awareness. PID5 said that the portrayal of a character with bipolar disorder in the soap opera “Eastenders” was particularly realistic. PID2 provided a link to the “Like Minds” television commercials in New Zealand that aim to reduce stigma and raise awareness of mental health conditions. PID6 remarked that he wished that British television would screen similar commercials. He related that he makes light of the illness through humor in the hope that others may accept mental health problems without fear or prejudice.

### Social Support

Participants sought advice and support from others via the forum, as well as providing it. Some participants invited others to coffee mornings and self-help groups organized by the Manic Depression Fellowship charity. Some participants revealed difficulties in communicating with family members about their bipolar disorder or struggling to rely on others in times of need. Other participants expressed their gratitude for having supportive families they could rely upon, and some acknowledged that their partners or children looked after them when they were ill. Responsibility was a key subtheme; when participants were very ill, they reported either relinquishing their responsibilities or feeling unable to.

PID3 commented on the importance of having social support, but lamented that she doesn’t feel comfortable with relying on others. PID1 said that her children have looked after her when she was incapable because of the illness, which gave rise to feelings of humiliation, shame, and guilt.

### Coping Strategies

Participants shared their personal coping strategies for dealing with boredom, staying well, and managing personal relationships. Exercise, routine, sleep, and diet were mentioned most frequently.

Some participants cited the importance of a regular sleeping pattern as a coping strategy. Those who worked shifts felt that this contributed to their becoming unwell. PID7 who worked shifts, reported drinking alcohol after a late shift and waking up at intervals throughout the night.

Some participants stated the importance of exercise, either to burn off excess energy or to improve low mood. The responsibility of being a member of a sports team motivated PID3 to reliably engage with her sports practice even when becoming ill. The discipline of this regular commitment to exercise enabled her to cope when she lacked energy. This participant also recommended writing things down in diaries, lists, or letters to release pressing thoughts and regain focus. Listening to music was another coping strategy cited by this participant, who said her mood could be affected by it, either to induce calm or excite. PID2 also reported lifting his mood through listening to music.

Some participants stated that engaging with the routine of work was their best coping strategy. Others mentioned that they tried to maintain a healthy diet, but struggled with their cravings for unhealthy, sugary food.

Regarding coping strategies for managing personal relationships, PID7 mentioned using code words with his partner to nonaggressively communicate warning signs of the illness. PID10 was wary of exposing herself to the emotional distress of others, such as a crying baby, her daughter’s emotional outbursts, or televised aggression.

### Insight and Acceptance

Through a greater personal understanding of bipolar disorder, some participants reported their increased self-esteem and a greater acceptance of the illness. Some participants commented that the program helped them gain insights into themselves and the trajectory of their illness.

PID4 remarked that she thought she had a good insight into bipolar disorder prior to the program, but has since learned new things and hopes to be able to accept the illness more. PID12 commented that he felt that he was learning more about bipolar disorder and could understand himself better.

PID13 said that she was finding the program and the forum to be very useful, despite her minimal contribution to the forum. She said that her episodes have become more seldom, she has made improvements to her lifestyle, and can now accept what she cannot change. She goes on to explain that now her employer and colleagues are fully aware of her condition.

PID1 said that she had recently begun to think of bipolar disorder as a problem with her neurotransmitters and a flaw in her make-up rather than a disorder with extreme moods or a mental illness.

### Life Chart

The life chart exercise was the most discussed aspect of the Beating Bipolar program, due to participants’ difficulties with completing it. Participants felt that it was too simplistic, and they needed to be able to add labels and notes regarding what medication they were taking and what triggered their highs and lows. Participants also needed the chart to begin before age 15 (if they felt that their bipolar disorder began at an earlier age), include the option to report a combination of medication, enable mixed episodes, and rapid cycling to be represented graphically, to show age at each point along the timeline, and to be able to select individual months or seasons. Some reported that the life chart was useful for explaining their illness to others and remembering events in greater detail.

PID11 commented that he was finding it difficult to remember events, especially when highs and lows occurred around the same time. He suggested that it would be helpful if he could draw a wiggly line with the mouse.

PID7 participant said that it would be useful if one’s exact age could be shown within a box that would appear as the cursor hovered over each point on the timeline. PID4 requested to be able to specify months within the timeline because her mood corresponded with the yearly seasons.

Some participants complained that they were not able to note on the chart when they were taking multiple medications at any one time. Which single medication to record or which mood to record if one’s moods were changing rapidly were also issues discussed, and PID8 struggled to record mixed states or periods of rapid cycling.

### Negative Experiences of Health Care

Participants described their negative experiences of health care. Some participants who had initially been misdiagnosed revealed the implications of their misdiagnosis for obtaining appropriate treatment, experiencing severe relapses, and employment.

A general practitioner (GP), who consequently referred him to a psychiatrist, diagnosed PID11 as having bipolar disorder. The psychiatrist refused to provide a diagnosis of bipolar disorder based on a single manic episode and refused to prescribe the medication that was previously prescribed to him by his GP. It took seven years before this patient received a diagnosis of bipolar disorder from another psychiatrist, who recognized his mania developing.

PID7 had received misdiagnoses from GPs, and had consequently taken medication that exacerbated the illness, until a psychiatrist reluctantly gave him a diagnosis of bipolar disorder. Due to his diagnosis, he had to retire from his career, and has since struggled to gain employment.

Participants highlighted difficulties in accessing a psychiatrist and a lack of continuity of care. Some related their experiences of doctors not listening to their concerns about medication or diagnosis, or doctors criticizing them for independently researching their illness. Some also felt that medical practitioners should increase their knowledge and understanding of bipolar disorder.

PID2 related that with the support of a good mental health team, many people with bipolar disorder could lead happy, healthy, and productive lives.

### Participants’ Use of Language

#### Personal Narratives

Participants frequently provided personal narratives relating to their history of bipolar disorder, life experiences, and backgrounds. These narratives were often confessional and contained anecdotes, metaphors, emotive language, and humor. Participants typically used a narrative style when describing their experiences with health care professionals, medication, and relationships with others. For example, participants would tell their story about how they came to be diagnosed with bipolar disorder, and how they came to be on their current medication, or would relate their story of their careers and how they came to be unemployed or retired as a result of the condition. Many of these narratives did not explicitly invite comments or advice from others; they appeared to be stories offered for the sake of sharing.

#### Humor

Participants used humor frequently within their posts. Humor was used for self-depreciation, irony, or sarcasm, and some participants used abstract or surreal metaphors to amuse. Many emoticons, abbreviations, and colloquialisms were also used.

PID8 joked that while his family sits down to have their cereal in the morning, he has a bowlful of antipsychotic and antidepressant medication. Another participant referred to the implications of his weight gain (which resulted from the side effects of his medication) on finding a girlfriend. With humor he remarked that not many women want to date an overweight man.

Some participants used metaphors that related to their perceptions of their careers or job prospects being worthless or discarded. Participants wrote of their careers “being binned” or having “fallen apart”. A couple of participants consequently regarded themselves as being “on the scrapheap” or “scrapheaped”. PID1 said that she felt as though her brain was “rotting quietly away” with lack of use.

Some participants illustrated happy or miserable smiling faces, following their own comments of a confessional nature. For example, PID3 disclosed that a significant problem of hers was an eating disorder. She ate to cope with emotions and regarded her eating to be an addiction. She revealed that after years of trying to overcome her eating disorder she has been unable to “break the cycle :( :( :(”.

Abbreviations used included “CPN” for “community psychiatric nurse”, “BD” for “bipolar disorder”, and “LOL” for “laugh out loud”.

### How Participants Engaged With Each Other

Participants shared their experiences via the forum and engaged with each other in a respectful manner. Some commented on others’ posts and some provided stand-alone narratives. Participants regularly sought and offered advice, and expressed encouragement and empathy. Some participants invited others to contribute to topics or to meet face-to-face. Links to external resources were also provided within some posts.

## Discussion

### Principal Findings

Only half of the participants contributed to the forum and only four participants contributed regularly, which suggests that the forum lacked the impetus for participants to continue to contribute, despite some input from DS. Participants used the forum to share and discuss what was relevant for them, to seek and offer advice, and to offer suggestions for improving the program. Posts were often personally revealing, yet, at the same time, usually carefully considered. Participants were respectful of each other, and their suggestions were often constructive and given in a supportive way.

The main themes which emerged from the forum posts were: issues regarding medication and employment, stigma, social support, coping strategies, insight and acceptance, the life chart exercise, and negative experiences of health care. Participants also provided personal narratives of their experiences, which often contained emotive language and humor.

Participants’ experiences of the forum, their reasons for not contributing, and their suggestions for its improvement were explored within one-to-one qualitative interviews [2]. Key observations from these interviews were: the lack of critical mass within the forum for worthwhile conversations, feeling put off by contributors who dominated topic threads, requiring reminders to log in regularly, and needing more input from health care professionals for new topics for discussion [2].

Since the publication of the BIPED trial [2,16], another randomized controlled trial has been published regarding the effectiveness of an Internet psychoeducation program for people recently diagnosed with bipolar disorder by Proudfoot et al [26]. This RCT was conducted in Australia and examined whether online peer support provided during the program affected participants’ symptoms and perceived control of their illness [26]. The authors developed an Internet psychoeducation program (Bipolar Education Program), which consisted of 8 weekly modules of 30-40 minutes in duration, encompassing the following topics: causes of bipolar disorder, diagnosis, medication, psychological treatments, omega-3, well being plans, and support networks [26]. There were 407 participants that were allocated at random to receive either an 8-week Internet psychoeducation program, an 8-week Internet psychoeducation program plus email support from expert patients, or weekly emails containing links to simple information about bipolar disorder [26]. Regarding the impact of peer support by email from expert patients, those who received Internet peer support had greater adherence to the program than those who did not [26]. This finding reflects the importance of peer social support, as identified in the present study, and the recommendation from Beating Bipolar participants who said that more input from a psychiatrist to the online forum may generate more engagement from participants within the forum, which lacked sufficient and regular contributions, thereby providing a greater opportunity for peer support [2].

An embedded qualitative study within the aforementioned RCT by Proudfoot et al [26] explored the email correspondence between the expert patients who provided the Internet peer support and those undertaking the Internet intervention and interviews with the expert patients [27]. They found that the informed peer supporter offered social comparison and experiential knowledge to the supported person and the peer supporter also received a greater sense of their own competence in managing their health as well as reciprocated peer support. Similar to participants of the present study, those newly diagnosed felt less stigmatized and isolated with the condition, and realized that “I’m not the only one!” and “other people experience this too!”. The expert patients in the Proudfoot et al trial offered empathy and practical advice which was grounded in their experiential knowledge, as well as enabling social comparisons to motivate and give hope to those newly diagnosed [27]. These elements of constructive peer support were also noted in the Beating Bipolar forum, as participants shared their experiences and offered advice and friendship.

A qualitative focus group study by Todd et al was designed to inform the design of an Internet-based self-management intervention (“Living with Bipolar”) for bipolar disorder by identifying the needs and desires of its prospective service users [3]. Participants stated the importance of techniques to manage their mood and also their lives more generally and said that the Internet is the only format which is freely accessible, instant, and interactive [3], a sentiment which participants of the BIPED study also shared [2]. The authors also suggest that professional and peer support may overcome low motivation [3], which echoes the qualitative findings from the RCT by Proudfoot et al [27]. The “Living with Bipolar” intervention comprised ten interactive modules, and participants were encouraged to use the online discussion forum to enable discussion of the modules and peer support [28]. The authors found that those participants who engaged with the forum completed more modules than those who chose not to engage with it, and everyone who completed the program used the forum in some way [28]. In the present study, we examined the nature of participants’ involvement in the online bipolar forum from the posts they shared, and witnessed advice giving, confessional narratives, and discussion of module topics, which is consistent with the peer support function of the “Living with Bipolar” forum [28].

### Strengths and Limitations

The way patients use self-help forums for bipolar disorder is an underresearched area. This study offers insights into how patients used the forum, topics which they feel are relevant to them following an education program for bipolar disorder, and how they interact with each other within an online community. The methodological approach of virtual participant observation is less obtrusive than interviews and has provided insights into how these patients shaped this online culture. By incorporating aspects of discourse analysis, the study revealed how participants commonly used humor in the form of metaphors or emoticons to convey emotionally sensitive issues and used a narrative style to self-disclose their personal stories to others.

Had there been more contributors and contributions to the forum, this study would have had a richer dataset on which to draw conclusions. Another weakness of this study is that we had not obtained consent from participants to use quotes from the forum. Unfortunately, it was not feasible within the scope of this research project to obtain the necessary consent in retrospect. A more in-depth discourse analysis may have also considered language structure, such as sentence length or word position [23], however this level of detail was considered to be beyond the scope of this study.

### Comparison With Prior Work

The predominant topic of medication within this forum was also one of the most discussed topics within studies of two German language forums for patients with bipolar disorder [1,14], which also cited patients’ social networks and symptoms as key topics. The studies inferred that participants’ main interest in contributing to a forum for bipolar disorder was to share emotion, as they identified disclosure, empathy, and support to be the main self-help mechanisms [1,14]. In our study, we identified much use of emotive language within participants’ narratives, as well as humor. It may be that participants’ frequent use of humor enabled them to communicate personal, emotionally charged issues in a less intense way, thereby diffusing any awkwardness and facilitating ongoing social interaction.

Regarding the use of emoticons in online forums, previous research has found that individuals “become” the text they write and the use of emoticons and expressive or “messy” texts can intensify interaction and push the boundary of what is possible in a textual conversation [21]. Participants’ use of humor, emoticons, and abbreviations formed their social “netiquette”, textual conventions which were to be adhered to in order for participants to “fit in” with their online community [17].

Other studies of online forums have also found the exchange of information to be a key feature [29-31]. In an ethnographic study of an online forum for obese and overweight people, researchers found that users exchanged a lot of information, including exercise tips, diets, and progress reports, alongside discussions of a weight-loss drug and its side effects [30]. Similarly, we found that patients exchanged much information relating to their coping strategies for dealing with bipolar disorder, and discussed exercise and dieting alongside other coping strategies such as the importance of maintaining a routine and a regular sleeping pattern.

A qualitative study of problems reported on an online depression support forum based in Australia presented six broad themes: “understanding depression”, “disclosure and stigma”, “medication”, “treatment and services”, “coping with depression”, and “comorbid health problems” [32]. Akin to our finding that participants expressed their reluctance to confide in colleagues and their fear of the consequences of self-disclosure, this study also revealed these concerns; however, the study also noted participants’ self-stigmatization, participants blaming themselves for their condition, and considering it to be a personal failing [32]. The BIPED forum did not present such self-stigmatization, perhaps because the forum was delivered as part of a psychoeducation trial and its participants had greater insights into their illness. Another finding of the depression forum study revealed an insight into participants’ reservations regarding seeking information from health care professionals; professionals may be perceived to lack the necessary skills or knowledge, lack sufficient time or be unavailable, and patients may fear a negative interaction with them [32]. Our study similarly highlighted participants’ negative experiences of health care, such as difficulties in accessing a psychiatrist and doctors not listening to their concerns about medication or criticizing them for researching their illness. These prior negative experiences may lead patients to seek information and support from nonmedical sources, as they may expect empathy, respect, and knowledge from patient support groups and forums.

Our finding that only 13/24 participants (54%) contributed at least once and only 4/24 participants (16%) contributed regularly to the forum highlights the discrepancy between participants who wish to merely observe an online forum and those who wish to actively participate in it. Within the BIPED semistructured qualitative interviews, participants were asked to comment on their experiences of the forum, and their reasons for engagement and nonengagement were explored [2]. From the qualitative interviews, we found that those who only observed the forum without contributing commented that they felt too self-conscious, were unfamiliar with online forums or insufficiently computer literate, or did not wish to engage with others who have bipolar disorder in case it triggered their low mood [2]. Forum users also remarked that more input from medical professionals was needed to contribute to discussions and start conversations by asking specific questions [2]. Furthermore, many appreciated the anonymity of Beating Bipolar and required privacy to log in; hence, public computers at libraries or hospitals were deemed to be unsuitable learning environments [2].

### Conclusions

Internet-based psychoeducation is a more private experience than face-to-face group psychoeducation for bipolar disorder, and in some instances may present less scope for enhancing social support. It may be most beneficial to those who lead busy lives, who are newly diagnosed, or who are disinclined to socialize with others in the context of a group health care program [2]. Online forums may be a cost-effective and pragmatic option for enhancing peer support for people with bipolar disorder, especially if provided in conjunction with an Internet-based psychoeducation program. They may provide patients with the opportunity to share their experiences and disclose and explore issues related to their illness anonymously. Although 13 of 24 participants in the intervention arm of the BIPED trial contributed to its forum, only four contributed on a regular basis. This forum would have benefitted from many more regularly contributing users to offer a greater support network with more diverse views and experiences. Further research is needed to explore how to optimally engage patients in using online health care forums.

## Abbreviations

BIPED: Bipolar Interactive PsychoEDucation trial
CMC: computer-mediated communication
GP: general practitioner
PID: Participant identification number
